# Towards a comprehensive set of GPS-based indicators reflecting the multidimensional nature of daily mobility for applications in health and aging research

**DOI:** 10.1186/s12942-019-0181-0

**Published:** 2019-07-24

**Authors:** Michelle Pasquale Fillekes, Eleftheria Giannouli, Eun-Kyeong Kim, Wiebren Zijlstra, Robert Weibel

**Affiliations:** 1 Department of Geography, University of Zurich, Winterthurerstrasse 190, 8057 Zurich, Switzerland; 2 University Research Priority Program “Dynamics of Healthy Aging”, University of Zurich, Andreasstrasse 15, 8050 Zurich, Switzerland; 3 Institute of Movement and Sport Gerontology, German Sport University Cologne, Am Sportpark Müngersdorf 6, 50933 Cologne, Germany

**Keywords:** Healthy aging, Conceptual framework, Spatial activity, Mobility indicator, Real-life assessment, Dimensions of mobility, Ambulatory assessment, Classification, Multi-dimensional

## Abstract

**Background:**

GPS tracking is increasingly used in health and aging research to objectively and unobtrusively assess individuals’ daily-life mobility. However, mobility is a complex concept and its thorough description based on GPS-derived mobility indicators remains challenging.

**Methods:**

With the aim of reflecting the breadth of aspects incorporated in daily mobility, we propose a conceptual framework to classify GPS-derived mobility indicators based on their characteristic and analytical properties for application in health and aging research. In order to demonstrate how the classification framework can be applied, existing mobility indicators as used in existing studies are classified according to the proposed framework. Then, we propose and compute a set of selected mobility indicators based on real-life GPS data of 95 older adults that reflects diverse aspects of individuals’ daily mobility. To explore latent dimensions that underlie the mobility indicators, we conduct a factor analysis.

**Results:**

The proposed framework enables a conceptual classification of mobility indicators based on the *characteristic* and *analytical aspects* they reflect. Characteristic aspects inform about the content of the mobility indicator and comprise categories related to *space, time, movement scope*, and *attribute*. Analytical aspects inform how a mobility indicator is aggregated with respect to *temporal scale* and *statistical property*. The proposed categories complement existing studies that often underrepresent mobility indicators involving timing, temporal distributions, and stop-move segmentations of movements. The factor analysis uncovers the following six dimensions required to obtain a comprehensive view of an older adult’s daily mobility: extent of life space, quantity of out-of-home activities, time spent in active transport modes, stability of life space, elongation of life space, and timing of mobility.

**Conclusion:**

This research advocates incorporating GPS-based mobility indicators that reflect the multi-dimensional nature of individuals’ daily mobility in future health- and aging-related research. This will foster a better understanding of what aspects of mobility are key to healthy aging.

**Electronic supplementary material:**

The online version of this article (10.1186/s12942-019-0181-0) contains supplementary material, which is available to authorized users.

## Introduction

Promoting healthy aging has become a key research endeavor by reason of increasingly aging societies around the world [1]. Mobility has been found to be an important predictor for individuals’ health and well-being, especially for older adults [2–6]. Key components of healthy aging such as well-being, social participation and active living are associated with different aspects of an individual’s daily mobility [7, 8]. Mobile individuals are able to access resources, which contributes to subjective well-being by making them feel independent [4, 9, 10]. Traveling using active modes of transport (such as walking or cycling) represents active lifestyles and is correlated with physical health and well-being [11, 12]. Moreover, the number of visited locations has been shown to be associated with social network size [13].

For the purpose of this work, daily mobility is a concept that describes the everyday spatiotemporal patterns of an individual’s movement in their environment [14]. Intertwined components of mobility are the spatial structure, the temporal structure, and the nature of activities [15]. Daily mobility is a key determinant for environmental exposure and access to resources as it defines when, where and how people are exposed to different environments (e.g., physical and social environment) [14, 16–18].

An individual’s daily mobility can be measured in different ways. Traditionally, mobility has been assessed subjectively and retrospectively via self-reports asking participants about their daily mobility behaviors (e.g., the life-space assessment [19] or travel diaries [20]). More recently, mobility has also been assessed using interactive map-based questionnaires (e.g., VERITAS tool [7]) and an increasing number of studies rely on passive location sensing methods—most prominently the Global Positioning System (GPS) [21]. Participants are equipped with such sensors embedded in either custom-built devices or smartphones that track a person’s locations in their natural environment in an objective, continuous, and unobtrusive manner [22]. GPS data can be used as input data to calculate *mobility indicators* that describe an individual’s daily mobility patterns. An indicator is defined as a measurable variable thought to be associated with a latent dimension (the true thing of interest, but not measured or unmeasurable) [23]. In this work, a mobility indicator is a variable that quantitatively describes an aspect of an individual’s daily mobility. For instance, commonly used mobility indicators include time out of home (TOH), number of trips, or size of life space [2, 24, 25].

**Fig. 1:**
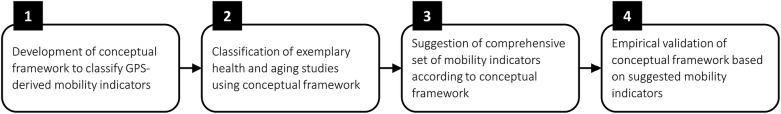
Workflow of this paper, leading towards a comprehensive set of GPS-based indicators. Squares represent the four sequential steps of the workflow

While many health and aging studies rely on only a few mobility indicators that illustrate partial facets of an individual’s daily mobility [2, 3, 26, 27], it has been increasingly emphasized that mobility is a multi-dimensional construct [22, 28–32]. There have been a few attempts to categorize mobility indicators [22, 25, 28, 33]. However, there is still little work on establishing a classification framework that groups and characterizes a wide range of GPS-derived mobility indicators according to spatial, temporal, and semantic aspects. Even with such a classification framework, it is still not obvious how differently similarly classified mobility indicators behave, and which groups of similarly behaving indicators exist. To respond to such limitations of existing studies, this paper takes a more comprehensive perspective on GPS-derived mobility and its multiple dimensions and therefore performs the following four steps, which also constitute our main contributions (Fig. 1):

- First, we establish a comprehensive conceptual framework, whose categories reflect different aspects of mobility and therefore enable categorizing and classifying GPS-based mobility indicators commonly used in health and aging research (“Classification framework for mobility indicators” section).
- Second, we employ the proposed framework to classify GPS-derived mobility indicators used in an exemplary set of health/aging studies, hence gaining insights into the aspects of mobility that are potentially underrepresented in the literature (“Classification of exemplary health and aging studies” section).
- Third, we demonstrate how the conceptual framework can be used to generate a comprehensive set of mobility indicators (“A comprehensive set of mobility indicators” section).
- Fourth, we conduct an exploratory factor analysis (EFA) to explore the latent dimensions of the proposed comprehensive set of mobility indicators. To do so, we compute the proposed mobility indicators based on 1 week of GPS data of a sample of 95 community-dwelling older adults (“Empirical validation of latent mobility dimensions: methods” and “Empirical validation of latent mobility dimensions: results” sections).

This research contributes to healthy aging research that involves real-life (spatial) mobility assessment, as it reflects the breadth of mobility aspects that are derivable from tracking data. Moreover, enhanced knowledge of the latent dimensions of mobility will help researchers to gain a more comprehensive view of an individual’s mobility and how its different facets differentially relate to health outcomes.

## Classification framework for mobility indicators

Based on its properties, each mobility indicator can be assigned to multiple thematically grouped categories that represent characteristic or analytical aspects. Characteristic aspects represent the actual semantic properties of the mobility construct; daily mobility can be described in terms of its spatial and/or temporal perspectives and can potentially be enriched with further attributes. Analytical aspects are not essential for the description of the content of the mobility construct per se, but rather refer to the processing of an indicator in terms of aggregation and statistical summary. The exact aggregation and/or summary methods used are dependent on the available data and the purpose of the study they are used for.

**Fig. 2:**
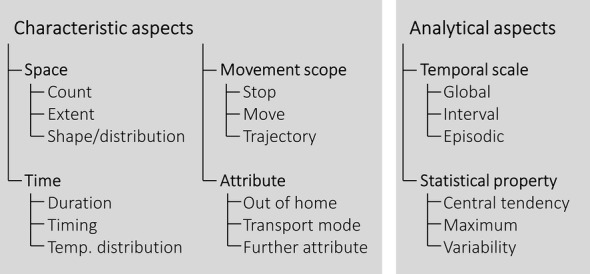
Proposed conceptual framework used to classify mobility indicators based on their analytical and characteristic aspects, which are then grouped into further thematically organized categories

The framework presented in Fig. 2 has been extended from the first attempt towards a classification framework described in Fillekes et al. [30]. In the following sub-sections, all categories are explained in detail.

### Characteristic aspects

Characteristic aspects provide information on the actual content of a mobility indicator. Mobility indicators are classified by one or multiple ***space*** and ***time*** categories. Moreover, they are grouped by one or multiple ***movement scope*** categories, each of which potentially can be enriched by further ***attributes***.

#### Space

The group of *space* categories summarizes indicators referring to different characteristics primarily inferable from the spatial distribution of the GPS data. The category ***count*** refers to the number of mobility-related events (e.g., the number of visited locations), whereas the category ***extent*** refers to the spatial size of the mobility-related activities. Extent can be measured using many different indicators including distance travelled or various types of ‘life-space’ indicators (in the spatial sciences also referred to as ‘activity-space’ indicators) [2]. Life space refers to the area within which a person moved over a specific period of time and is approximated using, for example, the convex hull or standard deviational ellipse based on the entire GPS trajectory [2, 34]. To answer health-related questions, it is meaningful to distinguish between counts and extent, as they do not necessarily correlate; an urban dweller, for instance, might cover a large life space in their day-to-day activities, but still might visit only a few locations due to the opportunity-sparse nature of their residential city. To assess to what degree an individual pursues an active lifestyle, which is an important healthy aging outcome [32], the latter might be more determining. The third category comprises mobility indicators referring to the ***shape/distribution*** of the location data (e.g., circularity of life space, or mono- vs. polycentric life spaces [31]). A polycentric life space could be interpreted as a more complex mobility pattern, which in turn could be related to higher levels of cognitive functioning [24].

#### Time

The categories related to *time* refer to different aspects regarding the temporal dimension of mobility patterns. The ***duration*** is the temporal aspect most commonly described by mobility indicators (e.g., time out of home, time spent in different transport modes). Spending time out of home or in different types of transport modes involves certain levels of physical, cognitive, and/or social activities and could therefore be related with an individual’s health status [35]. The category ***timing*** reflects the time of mobility-related events, which possibly indicates circadian or weekly patterns (e.g., peak of spatial activities in the morning vs. evening, or during week vs. weekend days, respectively). As an example, Shoval et al. [36] found that older adults with cognitive impairments would concentrate their out-of-home activities more in the morning and would also spread them less over the day. Eventually, the category ***temporal distribution*** comprises mobility indicators describing how time is distributed over different mobility-related activities. An exemplary indicator would be the entropy in visited locations. Having a low entropy (i.e., spending most of the time in few locations) has been found to be negatively correlated with depressive symptom severity [37].

#### Movement scope

The *movement scope* informs whether an indicator is based on the *stops*, *moves* or the mobility patterns engrained in an individual’s overall *trajectory*. Trajectory segmentation into ***stops*** (i.e., visited locations) and ***moves*** (i.e., trips between the locations) is an essential step when analyzing GPS data [38–40]. This process normally precedes enriching each segment with further attributional aspects (e.g., transport mode) that are presented in the subsequent *attribute* categories (next section). Indicators can then be used to separately describe stop or move segments. Stops are typically defined by a minimum time duration that an individual spent within a maximum radius (typically 30–150 m) [41–43]. In order to separate short—in a health context insignificant—stops (e.g., traffic light stops that can be seen as part of a move) from long significant stops (e.g., shopping, visiting friends, etc.), minimum stop durations between 5 and 15 min are commonly applied [44]. *Stops* are an approximation for the number of activities an individual performs and have been found to be positively associated with cognitive abilities [22]. *Moves* can be analyzed with respect to travel distances and transport modes used. Mobility indicators based on an individual’s exhaustive spatiotemporal footprint (i.e., all GPS points independently of the stop-move segmentation) are grouped in the category ***trajectory***. In movement analysis, a trajectory is defined as a sequence of successive positions of a moving object (in our case a human being) over a specific period (e.g., a day or a week) [45]. Mobility indicators assigned to this group comprise all GPS data including locations visited and routes travelled in between. Size of life space or time out of home are commonly used mobility indicators that would be assigned to this last category, as they are indifferent of a preceding move-stop differentiation of the trajectory.

#### Attribute

Some mobility indicators represent more semantic, qualitative, or nominal *attributes* of an individual’s mobility patterns as a whole or as a particular component (cf. movement scope) than the more basic spatiotemporal physical characteristics. In health studies, it is very common to quantify the number and duration of ***out*-*of*-*home*** activities [22]. Also, ***transport mode***, for instance, the distinction between active (non-motorized) and passive (motorized) modes, has relevance in health research. For example the duration of traveling using active transport modes is a proxy for transport-related physical activity [46, 47]. Depending on the research questions and data availability (e.g., additional self-reported information, GIS layers etc.) ***further attributes*** of an individual’s mobility may be quantified. Stops may be further semantically annotated based on performed activity types (shopping, health care, etc.). Trips may be annotated with their purpose or information about social interactions along the way [48]. Furthermore, exposure to environments (e.g., natural or physical environment) might be derived by combining individuals’ location data with different context information. In this paper, however, we focus on mobility aspects that are derivable from GPS data only.

### Analytical aspects

Each mobility indicator can be classified according to its ***temporal scale*** and ***statistical property***. Both groups of categories relate to properties regarding the level of processing. In principle, all mobility indicators could be computed to represent all of the temporal scales and statistical properties presented in the framework. Relevant temporal scales and statistical properties used to aggregate the mobility indicators are defined below. The decision which temporal scales and statistical properties are actually used depends on the available data and purpose of the particular study that is carried out.

#### Temporal scale

The following temporal scales can be used to aggregate and summarize mobility indicators depending on available data and purpose of analysis: *global, interval,* and *episodic* [49]. At the ***global*** scale, mobility indicators of each individual are aggregated over the entire study period (e.g., 1 week, 1 month). Studies based on the global scale typically investigate how individuals’ daily mobility patterns relate to their overall health outcomes using cross-sectional study designs [34]. In health research, a study period to assess an individual’s daily mobility behaviors is often 1 week [7, 26, 50, 51]; in this case, the global mobility indicator aggregates 1 week of GPS tracking into a single value. As an example, Takemoto et al. [52] computed mean daily number of vehicle trips as a global mobility indicator (aggregated from 6 days of GPS tracking) and found negative association with fear of falling.

At the ***interval*** scale, mobility is assessed over multiple time periods (i.e., intervals; e.g., daily, hourly) dissected from the entire study period; mobility indicators are then aggregated over each interval. Interval-based assessments focus on within-person fluctuations in both mobility and health outcomes. Specifically, an interval-scale mobility indicator (e.g., daily travel distance for every study day) enables a longitudinal study design that examines questions such as associations between health outcomes and certain mobility indicators at the within-person level [53]. For instance, Kaspar et al. [27] investigated whether daily time out of home can predict a person’s daily mood.

At the episodic scale, mobility indicators are aggregated by ***episodes*** that are defined by an external criterion (e.g., weekend day, walking segments, or periods with high levels of subjective well-being) [54]. Saeb et al. [55], for example, computed mobility indicators for week- and weekend-days separately and found that the latter have stronger associations with depressive symptom severity. Moreover, indicators such as walking speed [24] reflect mobility characteristics of a particular episode—in this case a walking segment.

#### Statistical property

Mobility indicators can be classified according to several types of descriptive statistics. In a health context, mobility indicators reflecting *central tendency* (e.g., mean, median), *maximum* (e.g., maximum or 90th percentile), or *variability* (e.g., standard deviation, coefficient of variation) illuminate different perspectives of mobility and have different relationships with an individual’s health outcomes [26, 56]. While ***maximum*** indicators relate to the highest performance of the respective construct—which is also referred to as capacity—***central tendency*** indicators reveal the average behavior or, in other words, give insight into the extent to which capacity (in each construct) is exploited [57]. Maximum distance from home (as an example for a *maximum* indicator) was related to cognitive functioning—more specifically planning and attention [34] and memory [58]. Daily average GPS-derived out-of-home time (as an exemplary *central tendency* indicator) was found to correlate with physical functioning [59]. ***Variability*** indicators give insight into the regularity/diversity of an individual’s mobility-related behaviors [60]. While GPS-derived variability indicators have not been used often so far, several studies have calculated variability in physical activity using inertial sensor data and showed that *variability* indicators are very relevant for health and functioning, but not always as a positive association [56, 61].

## Classification of exemplary health and aging studies

In order to employ the classification framework introduced above and show what mobility aspects are typically represented by mobility indicators in health- and aging-related studies, we have chosen an exemplary set of papers and classified them based on the mobility indicators used according to our classification framework (Fig. 2). After a broad but non-systematic, non-exhaustive literature search focusing on health and aging studies involving GPS-based mobility indicators, we retained articles that utilized mobility indicators based exclusively on GPS data and where the indicators were related to health- and aging-relevant outcomes. Papers comprising self-reported questionnaire- or map-based indicators as well as exposure-related indicators and studies assessing the feasibility and validity of GPS indicators were excluded. Some of the included papers were also found in recent systematic reviews on sensor-based assessments in health [62–64]. Moreover, the GPS-based mobility indicators utilized in the studies analyzed in these review papers are very similar to the ones covered by the exemplary papers used in this article (cf. Table 2 and in Additional file 1: Table S1).

The selected studies were classified according to the mobility aspects that are covered by at least one of the included mobility indicators (Table 1). The detailed classification, assigning each indicator used per study separately to the categories of the proposed framework is shown in Additional file 1: Table S1. In Table 1, characteristic aspects represented by each study are shown by check marks. Analytical aspects are not included because little between-study variability was found with respect to the *temporal scale* and *statistical property* categories. Most of the studies use indicators aggregated to daily mean/median or weekly total values which reflect the *global* temporal scale and the statistical property *central tendency*.

**Table 1:**
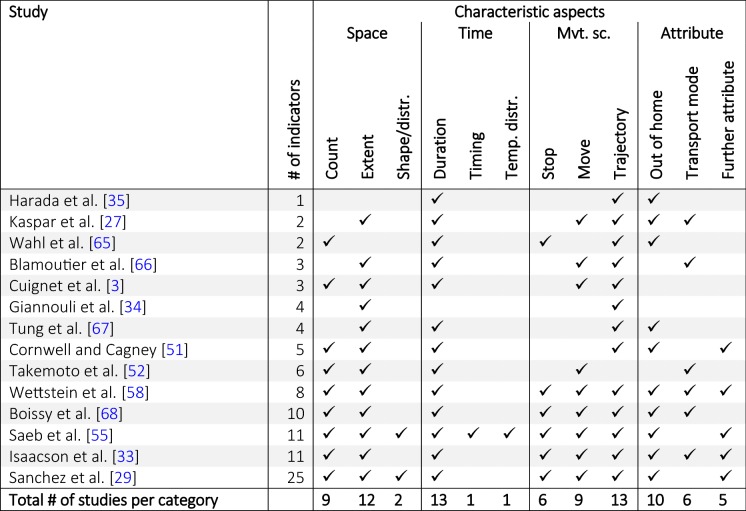
Allocation of exemplary health-/aging-related studies based on the GPS-derived mobility indicators used, according to the characteristic aspects of the classification framework of Fig. 2

‘Mvt. sc.’ is the abbreviation of ‘Movement scope’. For the detailed classification of the individual indicators Additional file 1: Table S1. Number (#) of indicators refers to the total number of GPS-derived mobility indicators that were included in the respective studies

Most of the studies are based on a relatively small number of indicators (often less than 6 indicators). Even the studies involving many indicators do not cover all the categories we suggest in our framework. For example, in the paper by Sanchez et al. [29] indicators reflecting *timing* or *temporal distribution* of activities as well as indicators characterizing the *move* scope are not considered. The most comprehensive set of mobility indicators according to our scheme is provided by Saeb et al. [55]. The only aspect they do not take into consideration is the *transport mode*.

The most frequently used space-related categories are *extent* (12  studies) and *count* (9 studies), while *duration* (13 out of 14 studies) is the most frequent time-related category. Categories referring to more qualitative aspects of space and time, such as *shape/distribution*, *timing* and *temporal distribution* are only covered by a minority of the investigated studies. With respect to the movement scope, most studies include indicators referring to the entire *trajectory* (13 out of 14). Around half of the studies involve indicators that are based on the pre-segmented trajectory into *stop* and *move* episodes. With respect to the attribute-related categories many indicators quantify the amount of *out*-*of*-*home* activities or distinguish between active and passive *transport mode*.

These observations are also confirmed on the level of the most frequently used mobility indicators (Table 2). Mobility indicators used in ≥ 2 studies are most often representing the categories *extent*, *count*, *duration* and most cover the movement scope of the entire *trajectory*. Only a few indicators have been dominantly used, many of them reflecting similar combinations of mobility aspects (e.g., TOH, maximum distance from home, standard-deviational ellipse, area of convex hull, or time in vehicle).

**Table 2:**
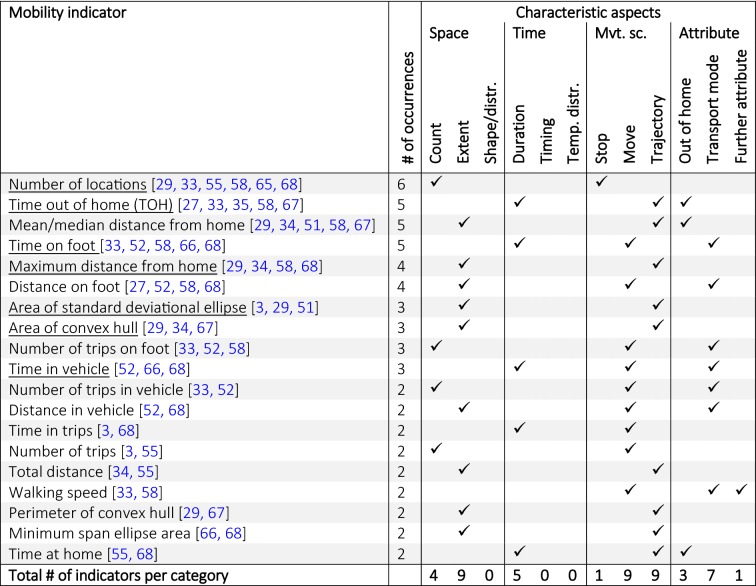
Mobility indicators used in at least 2 studies of the 14 studies listed in Table 1 according to the classification framework

Indicators underlined above were included in the suggested set of mobility indicators presented in the subsequent Table 3. This table was derived from the detailed classification of the individual indicators per study in Additional file 1: Table S1

## A comprehensive set of mobility indicators

We propose a set of mobility indicators (see Table 3) that—in contrast to the studies presented above—is comprehensive in the sense that all characteristic aspects of the above introduced classification framework are covered. Regarding the analytical aspects, in line with many other studies [34, 51, 59], we focus on a *global* temporal scale, i.e., all mobility indicators were summarized to one value reflecting the entire study period. Moreover, most of the mobility indicators were aggregated to daily average values for each participant, which reflects the statistical property *central tendency*. In the selection of mobility indicators, we assured that each characteristic category was represented by at least two indicators, so they could potentially load on a factor in the subsequent factor analysis (cf. “Empirical validation of latent mobility dimensions: methods” section) if they are capturing a sufficiently distinct underlying dimension of an individual’s daily mobility.

**Table 3:**
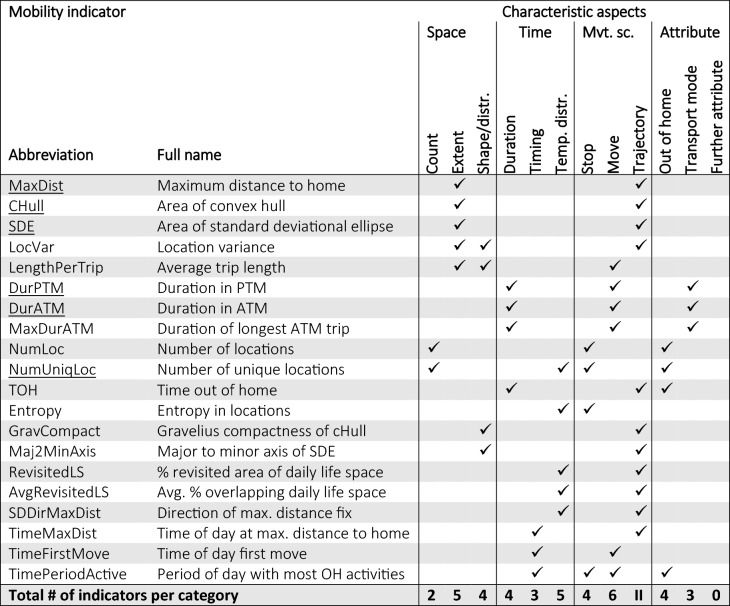
Proposed set of mobility indicators according to the classification framework of Fig. 2

Underlined indicators are amongst the frequently used ones according to Table 2. Abbreviations: passive transport mode (PTM); active transport mode (ATM), standard deviational ellipse (SDE), convex hull (cHull), out of home (OH). The computation of the indicators is described in Table 5

We included the majority of the most frequently used mobility indicators from the literature (see the mobility indicators underlined in Table 2 and Table 3) and complemented them with less common indicators or suggested by ourselves in order to ensure covering all the characteristic aspects of the classification framework. Complementary indicators identified from the literature include average trip length [33], entropy [55], location variance [55], maximum duration using active transport modes [69], number of unique locations [25], Gravelius compactness of convex hull, and major to minor axis of standard deviational ellipse [28]. The *timing*-related as well as the indicators representing the category *temporal distribution* are proposed by ourselves and—to the best of our knowledge—have not been used in any other mobility-related health and aging study. The precise definition of all included mobility is described further below (Table 5).

## Empirical validation of latent mobility dimensions: methods

In order to explore the latent dimensions of the set of chosen mobility indicators, we computed these indicators using GPS data from the ‘Mobility study’ of the German Sport University Cologne and subsequently conducted an exploratory factor analysis (EFA).

### Participant recruitment

The recruitment strategy and the study admission criteria are described in detail elsewhere [26]. In summary, community-dwelling older adults were recruited primarily by handing out information brochures and holding presentations about the study in local senior citizen gatherings. In total, 192 persons meeting the criteria for participation in the study were recruited. Study admission criteria were age older than 60 years, no serious diseases that could interfere with functional mobility, and the ability to stand up from a chair independently. All participants signed an informed consent form agreeing to participate in the study.

### Ambulatory assessment

Mobility performance in real life was assessed over approximately 1 week by means of smartphone technology. Each participant was given a smartphone (Samsung Galaxy SIII™), which they were asked to carry with them all day. Collection of GPS data was run in the background so that the only required interaction of the participants with the smartphone was to charge it at night. Data recording took place between the first appointment, in which participants received the smartphone and the second appointment, in which they returned it. The aim was to record the participants’ real-life mobility for 7 days. However, it was not always possible to organize the appointments exactly 7 days apart. As a result, the total registration time ranged from 6 to 9 days.

### GPS data processing and computation of mobility indicators

All processing and analyses of the GPS data were carried out in R (v. 3.4.4/3.5.2 [70]). Specifically, we used the R packages plyr, dplyr, reshape, sp, dbscan, data.table, aspace, geosphere, circular rgdal, and raster for data manipulation; ggplot2, maptools, knitr for graphs and visualizations; and Hmisc, PerformanceAnalytics, nFactors and corrplot for the statistical analyses.

#### GPS data processing

GPS data processing consisted of the following four steps: exclusion of outliers; splitting into daily trajectories; segmentation into stops and moves; annotation of moves based on transport mode; and annotation of stops as home/out of home and unique/multiply visited.

First, outliers were excluded by removing GPS fixes (an individual location point defined by its coordinates and an associated timestamp) with speed above 330 km/h, which corresponds to the maximum speed of high-speed trains in Germany. The weekly GPS trajectories were split into daily segments at 3 AM, similar as in Schneider et al. [71], assuming that participants would go to bed latest at 3 AM and therefore no uniform activities would accidentally be split in two.

Second, GPS points were segmented into move and stop segments using the algorithm suggested in Montoliu et al. [72]. A stop was defined as a geographic region (< 150 m) in which a participant stayed for at least 5 min and was represented by the position of the median latitude and median longitude of the included GPS fixes, and by the timestamps when the participant arrived and left the stop, respectively. If two consecutive location fixes within the same stop had more than one hour time difference due to a potential data gap, they were designated as two separate stops. If they did not lie within the same stop, the latter fix was labeled as *jump*. GPS fixes in between the identified stops that are longer than 3 min were designated as move segments, similar as in Vanwolleghem et al. [73]. Consecutive stops interrupted by short segments (i.e., ≤ 3 min) not identified as stops were merged in a second step, if the distance between stops was smaller than 150 m and the time interval shorter than one hour.

Third, segmented moves were classified into segments traveled with *active* (non-motorized) and *passive* (motorized) transport modes (ATM and PTM, respectively). Like in Carlson et al. [42] and Vanwolleghem et al. [73], move segments with 90th percentile speed ≥ 25 km/h were classified as *passive*, segments below this threshold as *active*.

Fourth, segmented stops were classified into home and out-of-home (OH) locations. Similar to Loebach and Gilliland [75], we used a buffer of 150 m around home to define GPS fixes or identified stop points as OH fixes or stops, respectively. Finally, we identified and marked stops visited multiple times (referring to the same location cluster). We used density-based clustering for this purpose, with an epsilon radius of 60 m and minimum number of stops of two.

#### Inclusion criteria

Study days were considered as valid using the following criteria:

- Regarding the daily minimum temporal GPS wear time to count as valid day, we tested three commonly used thresholds: 8 h [50, 66], 9 h [73, 74], and 10 h [35, 75].
- A day was excluded if no stop was identified [25].
- Finally, days including visits to the lab where the appointments for receiving/returning the smartphone took place, were excluded (i.e., no GPS fixes tolerated within 200 m of the lab on the first/last study day).

Following criteria were applied for participants to be included in the final analyses:

- Only those participants who had corresponding address-based and GPS-based home addresses (i.e., distance of less than 170 m between them) were included. GPS-based home location was computed by using density-based clustering based on the first morning and last evening fix of every valid day, identifying clusters with a minimum number of three fixes within an epsilon distance of 60 m, which corresponds to the average positional error in bad GPS reception condition [76]. The cluster closest to the address-based home was chosen as GPS-based home location. For further computation, we replaced the address-based home with the GPS-based home, in case the latter reflected more precisely the GPS fixes, i.e., more than 1% more fixes within a 60-m-buffer of the GPS-based than the address-based home.
- To represent typical daily mobility, we tested a minimum number of required study days of 3 [75], 4 [74], and 5 [66].
- One of the included days had to be a weekend day and the remainder of days had to be weekdays in order to achieve a representative view of an individual’s mobility over the course of an entire week, as the level of mobility has been found to be unequally distributed between week- and weekend-days [27, 77].
- At least 3 days (one weekend day, two weekdays) out of the valid days had to be days on which at least one move segment was registered.
- Three participants who reported non-habitual movement during the registration period in the post-study questionnaire were excluded.

Table 4 shows the resulting number of included participants fulfilling all the inclusion criteria and the following five input data conditions: 3 days/8 h, 4 days/8 h, 4 days/9 h, 4 days/10 h, and 5 days/10 h. Minimum duration of daily registration period was based on duration between first and last fixes of a study day independent of potential gaps in GPS data throughout the day (e.g., due to missing satellite signal). Such gaps are not necessarily problematic in measuring daily mobility because GPS signal loss mostly occurs in buildings and often such data loss is independent from the spatial extent of daily activities (e.g., the maximum distance from home). Moreover, for some indicators (e.g. time out of home) missing data can be interpolated with little risk for errors as detailed in the subsequent section.

**Table 4:** Number of participants meeting the aforementioned input data requirements, for different combinations of minimum number of valid days and minimum daily duration of registration period

n = days/x = hours	8 h	9 h	10 h
3 d	95	N/A	N/A
4 d	85	80	74
5 d	N/A	N/A	51

‘N/A’ means that this condition was not tested

#### Computation of mobility indicators

We computed the proposed set of daily indicators (Table 3) describing diverse aspects of an individual’s daily mobility. The definitions of the daily indicators and the aggregation to weekly indicators for a randomly selected subset of days consisting of 1 weekend day and 2, 3, or 4 weekdays, depending on the inclusion criteria are given in Table 5. For indicators that are only meaningful if there was some out-of-home activity throughout a day (e.g., timing-related indicators), we randomly selected 3 days, two out of the valid weekdays and one out of the valid weekend days, provided at least one move existed for each selected day (marked by ‘M’ in day selection; Table 5). In order to see whether the random selection of study days had an impact on the results, we computed 10 runs for each of the combinations of inclusion criteria listed in Table 4.

**Table 5:** Description of the computation of the mobility indicators

Mobility indicator	Day selection	Definition of daily mobility indicator
MaxDist	R	Length of straight line connecting the home with the GPS fix furthest away from home
CHull	R	Area of convex hull enclosing all GPS fixes
SDE	R	Ellipse defined at one 1 SD containing approximately 68% of GPS fixes within the ellipse’s boundary
LengthPerTrip	M	Average length of a move
LocVar	R	Combined variance of X and Y coordinates [55]
DurPTM	R	Time spent in passive transport modes
TOH	R	Duration between all OH fixes, interpolating for up to 60-min gaps between consecutive GPS fixes if both fixes are OH
Entropy	R	Entropy computed as in Saeb et al. [55]. Entropy measures how a participant’s time was distributed over the different stop locations: the higher the entropy, the more regularly time is distributed and/or the higher the number of unique locations
NumLoc	R	Number of OH locations visited
NumUniqLoc	M	Stops visited multiple times (referring to the same location cluster) during the included study days are only counted once
DurATM	R	Time spent in active transport modes
MaxDurATM	R	Duration of longest continuous trip using active transport modes
RevisitedLS	M	Percentage of the daily convex hull that has overlap with any convex hulls of the other included study days
AvgRevisitedLS	M	Average percentage overlap of the daily convex hull with the convex hulls of the other included study days
SDDirMaxDist	M	Direction of most distant point from home. Weekly aggregation is done by circular SD: the larger the circular standard deviation, the more variability in day-to-day orientation of life space
GravCompact	M	$K = P/(2\sqrt {\pi A} )$ (where P = perimeter of convex hull and A = area of convex hull. The higher the more elongated is the life space
Maj2MinAxis	M	Ratio between major and minor axis of standard deviational ellipse
TimeMaxDist	M	Time of day starting at 3 AM [min] when most distant location from home is reached
TimeFirstMove	M	Time of day starting at 3 AM [min] of the first move (approximation of first OH activity) of a day
TimePeriodActive	M	Assignment of OH activities (moves and OH stops) based on start time to the classes morning (6 AM–12 noon), afternoon (12 noon–6 PM), or evening (6 PM–11 PM). A day is coded as 1 (morning day) if morning activities > evening activities; as 3 (evening day) if evening activities > morning activities; 2 (neutral timing day) in all other cases

‘Day selection’ refers to whether among the valid days a fixed number of days were selected completely at random (R) or only if days included at least one move (M). All daily indicators were summarized to weekly aggregates using the median, except for SDDirMaxDist, where the circular SD was used, and TimePeriodActive using the mean

### Statistical analyses

What is the minimal set of mobility indicators that comprehensively reflect an individual’s daily mobility? One solution is to use only one indicator from each group of indicators that behave similarly. An exploratory factor analysis (EFA) [78, 79] is known to uncover latent groups of input variables (here: mobility indicators). In this manner, we can compress the comprehensive set of mobility indicators further into the minimal set of indicators required to represent the diverse aspects of an individual’s daily mobility derivable from GPS data.

We used the maximum likelihood factor analysis function (factnatal) from the stats package in R [70] with the orthogonal rotation method varimax. Statistical inference is improved if the variables are normally distributed. For non-normally distributed mobility indicators, we applied log and square-root transformation, respectively, based on their effectiveness to achieve a normal distribution or at least reduce skewness.

Given that there is no commonly accepted standard for GPS wear time [75] and in order to assess a potential effect of GPS data quality on the obtained results, EFAs for each of the aforementioned 5 input data conditions (in Table 4) were iteratively conducted 10 times (thereby varying the random sampling of study days) (50 runs in total). EFA requires designating the number of latent factors of input variables as an input parameter. To determine the optimal number of latent factors for each of 50 EFAs, we adopted four visual/non-visual estimation methods provided by the nScree function of the R package nFactors [80]: Kaiser rule, parallel analysis, optimal coordinates, and acceleration factor. The mode of the suggested numbers of optimal factors from four methods was used; if two modes appeared, the median of the numbers was used. We visualized EFA results collectively by using a pair matrix. The pair matrix sums up EFA results by counting the number of co-appearances of each pair of input mobility indicators in the same latent factor over multiple runs.

For the data condition 3 days/8 h, the cases-to-parameter (N:k) ratio with 4.75:1 (N = 95 participants, k = 20 variables) is below the ideal size-to-parameter ratio of 20, however, still above the minimal three observations per estimated parameter [79]. In order to assess the suitability of the input data for factor analysis, we performed the Kaiser–Meyer–Okin (KMO) Measure of Sampling Adequacy and Bartlett’s Test of Sphericity, which, in particular, are recommended when the N:k ratio is below 5:1. The obtained KMO index (which can range between 0 and 1) is 0.78 for the exemplary individual run of EFA presented subsequently in Table 7 and thus considerably higher than the minimum recommended 0.50; Bartlett’s test is significant on the level p = 0.05 [79]. Both tests thus indicate that the input data is suitable for factor analysis.

## Empirical validation of latent mobility dimensions: results

As we found the resulting factors to be fairly stable across the five input data conditions, we decided to present in this section solely the result for the condition with the least strict requirements on data validity (n = 3 days/x = 8 h) to maximize the number of included participants. The pair-matrix tables for the remainder of the data conditions are presented in the Additional file 1: Figures S2–S6.

### Included participants

For the selected input data condition, 95 out of the original 192 participants were included (Table 4). The overall attrition rate with 50.5% was rather high but comparable with other studies conducting GPS data collection over similar observation periods, e.g., 44% in [75], 48% in [50], and around 50% in [46]. Technical problems (storage, battery issues, mobile phone settings etc.) and participant compliance (not charging devices) are common issues that may lead to high attrition rates in studies based on real-life datasets. In contrast to technical problems that are expected to occur independent of participant characteristics, high attrition rates are a limitation for GPS-based studies due to a potential ‘selection bias’ [21, 81, 82]. Poor data quality might be caused by low participant compliance or certain mobility patterns (staying a lot indoors) that are related to low socio-economic status. However, a selectivity analysis, showed that in terms of socio-demographics the participants that were excluded due to inadequate data (n = 97) did not show any statistically significant differences to the included participants (n = 95) (p < 0.1 for gender, age, BMI, and education).

The 95 participants with valid GPS data had on average valid data for at least 8 h on 5.7 days. Mean age was 70.5 years (range 61–99), and 52% were female. Body mass index (BMI) was 24.7 ± 3.7, the majority of the participants (88 out of 95) suffered from at least one chronic disease, which is representative of this age group’s health status [83]. Most of the participants were pensioners but they varied in terms of their education level. Table 6 presents descriptive statistics for a selection of GPS-derived mobility indicators of the included participants.

**Table 6:** Mean, median, and standard deviation (SD) for a selected set of the median daily mobility indicators per participant, aggregated over the entire study population (n = 95)

Variable	Mean	Median	SD
Time out of home (TOH) [min]	174.0	157.0	114.1
Maximum distance to home (MaxDist) [km]	12.2	3.8	39.3
Area of convex hull (CHull) [km^2^]	74.5	3.8	617.5
Number of OH locations (NumLoc)	2.6	2.0	1.6
Duration in active TM (DurATM) [min]	42.5	33.9	42.8
Duration in passive TM (DurPTM) [min]	25.3	7.1	40.1
Percentage revisited life space (RevisitedLS)	0.5	0.5	0.3

### Factor analysis

Table 7 presents the results of the EFA for a selected run for the input data condition 3 days/8 h, yielding a solution of 6 latent factors (identified as the optimal number of factors based on 3 out of 4 statistical tests). Across all different runs and input data conditions the 6-factor solution was clearly the one most often suggested by the four statistical tests (see Additional file 1: Figure S1). The presented solution in Table 7 explains 68% of the overall variance. The p-value for the hypothesis that the model fits the data perfectly is 0.14 and *H*_0_ consequently cannot be rejected. We have labeled the factors based on the mobility indicators that load on them as follows: 1 = extent of life space; 2 = quantity OH activities; 3 = time spent in ATM; 4 = stability of life space; 5 = elongation of life space; and 6 = timing of mobility.

**Table 7:** Factor loadings for the set of mobility indicators listed in Table 5 to uncover latent mobility dimensions (for one out of the 10 EFA runs for the data condition 3 days/8 h)

Variable	Factor					
	1 Extent of life space	2 Quantity OH activities	3 Time spent in ATM	4 Stability of life space	5 Elongation of life space	6 Timing of mobility
**% explained variation**	22%	12%	11%	9%	8%	7%
MaxDist^b^	0.87					
CHull^b^	0.94					
SDE^b^	0.93					
LengthPerTrip^b^	0.70					
LocVar^a^	0.79					
DurPTM^b^	0.48					
TOH^c^		0.64				
Entropy^b^		0.76				
NumLoc^b^		0.83				
NumUniqLoc^b^		0.37				
DurATM^c^			0.96			
MaxDurATM^c^			0.96			
RevisitedLS^a^				0.95		
AvgRevisitedLS^c^				0.74		
SDDirMaxDist^a^				− 0.40		
GravCompact^b^					0.93	
Maj2MinAxis^b^					0.72	
TimeMaxDist^c^						0.74
TimeFirstMove^c^						0.65
TimePeriodActive^c^						0.51

Extraction method: Maximum-likelihood factor analysis. Rotation method: varimax. Transformations: ^a^original, ^b^log transformed, ^c^square-root transformed. Variables’ factor loadings are displayed for the factor that they correlated most with. Test of the hypothesis that 6 factors are sufficient cannot be rejected (p-value is 0.137). The six factors capture over 68% of the variance originally observed between the 20 variables

**Fig. 3:**
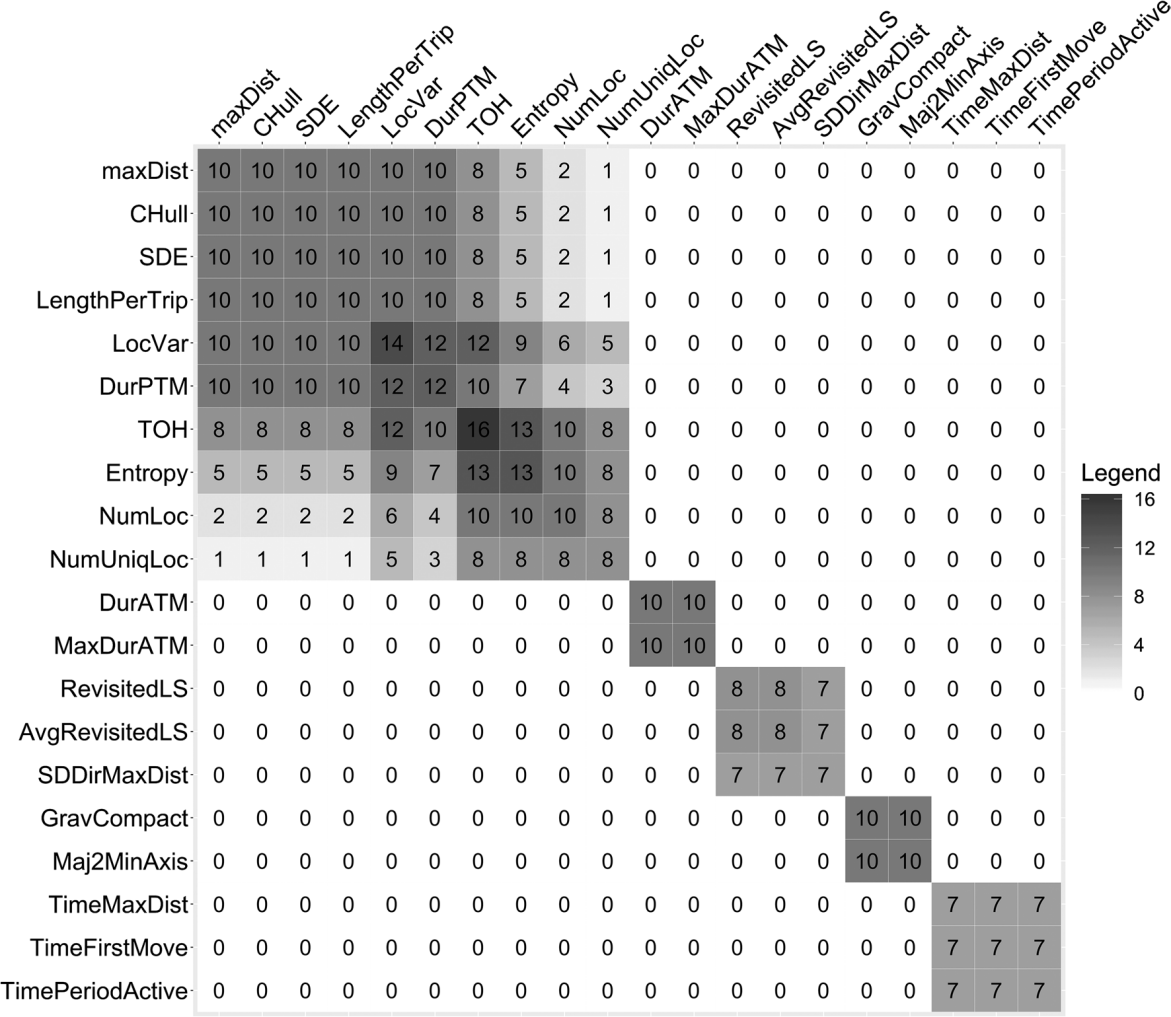
EFA summary matrix for the 10 runs of random day selection, using the inclusion criteria 3 days with at least 8 h registration period per participant. The counts indicate how often each pair of mobility indicators appears together in a factor with a minimum factor loading threshold of 0.4. The mobility indicators are ordered in the same way as in Table 7

In order to assess the stability of the results across the 10 different runs, the pair matrix visualization in Fig. 3 shows how often each pair of variables appears together in the same factor(s) for each run. The summary matrix visualizations of the remaining input data conditions can be found in the Additional file 1: Figures S2–S6. The EFA matrix shows that most of the mobility indicators seem to consistently co-appear throughout the different runs. For example, DurATM and MaxDurATM co-appears in all 10 runs of EFA. Not all mobility indicators are consistently assigned to the same factor, however. Indicators such as TOH, entropy, NumLoc, NumUniqLoc sometimes load on the extent of life space and/or the quantity of OH activities, respectively, suggesting that these two factors may be partially correlated.

Table 8 illustrates all the categories to which the mobility indicators that load on a factor have been assigned (based on the solution presented in Table 7). It shows that the different factors reflect different combinations of categories of the suggested framework. The first factor seems to consist of a mixture of factors reflecting the size of life space, variables describing spatial distribution, as well as time spent in passive transport modes. The second factor is about number, duration, or temporal distribution of OH activities (broadly the quantity of time that is spent out of home). Factor 3 seems to be composed only of variables reflecting quantities of traveling using active modes of transport. The stability of life space assesses the degree of overlap in the day-to-day spatial footprint. Factor 5 reflects the elongation of life space: the larger the factor the more elongated an individual’s life space. Factor 6, finally, informs about how late in the evening an individual is active out of home.

**Table 8:**
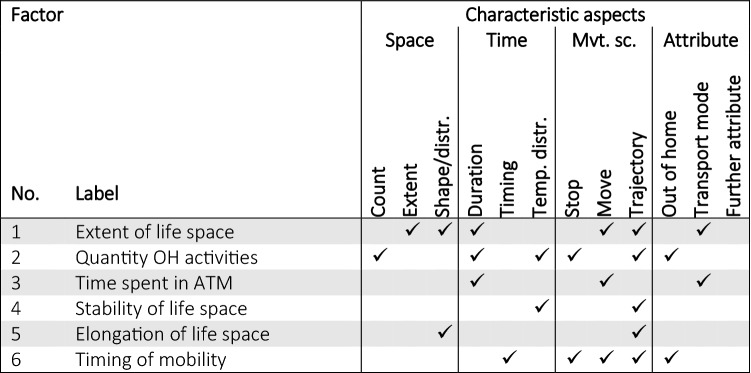
Factors assigned to the categories of the classification framework (Fig. 2) based on categorization of the mobility indicators that have their highest loadings on the corresponding factor

## Discussion

### Mobility indicator classification framework

The current paper presents a framework to classify GPS-derived mobility indicators according to a comprehensive set of distinct aspects of mobility (Fig. 2). We focused on mobility indicators derivable from GPS data because this is the current standard location sensing technology [21]. However, it is possible to apply the framework for indicators derived from other geolocation data sources including map-based self-reported data [17] or passively collected mobile phone network-based data, such as those stored for billing purposes [84].

The framework shows the breadth of aspects that can be derived from GPS-data and exhibits how mobility indicators can reflect different combinations of characteristic and analytical aspects. The explicit categorization of mobility indicators allows to conceptually understand which aspects of mobility are represented. By classifying existing papers that used GPS-derived mobility indicators according to the proposed framework, we found that more quantitative indicators reflecting *count*, *extent* and *duration* clearly are most often used, while indicators describing more qualitative spatial and temporal aspects (such as *shape/distribution*, *timing* and *temporal distribution*) are under-represented (Table 1). Additionally, most of the publications include indicators reflecting the entire trajectory while only half of them included indicators characterizing the movement scope of *stops* and *moves* separately, which was already observed by Chaix et al. [15].

A few studies have attempted to classify mobility indicators based on a priori hypotheses [22, 28, 29, 33]. However, they focused only on limited groups of indicators and do not allow for the categorization of a broad set of indicators. Brusilovskiy et al. [25] grouped mobility indicators into three themes: community participation (total number of locations), geographic scope of mobility (area), and temporal scope of mobility (TOH). The categorization of Wettstein et al. [22, 58] is similar, except for aggregating temporal scope and community participation to one class—global mobility (TOH, number of locations)—which in our opinion should be separated as they most probably are not related (loading on two different factors in our EFA). Similar to Brusilovskiy et al. [25] they also suggest a walking-related category (number of walking trips, etc.). The categories used in the mentioned studies [22, 25, 58] are equivalent to the following categories of our classification framework: *count*, *size*, *duration*, and *transport mode*.

A novelty of our classification framework is the distinction of mobility indicators describing *stop*- and *move*-episodes, or alternatively the entire *trajectory*. Moreover, our classification scheme allows for a classification of indicators related to *shape*, *timing* and *temporal distribution* of an individual’s mobility patterns. Finally, our classification framework is the first to include analytical categories that make explicit the *temporal scale* that an indicator reflects; and it emphasizes the different information transmitted by indicators reflecting the different statistical properties *central tendency*, *variability* and *maximum*. An extension of the proposed framework might include a characteristic category ‘space–time’, including indicators reflecting speed and acceleration, highlighting further distinct aspects of mobility derivable from GPS. Walking speed, for example, has been found to be a major indicator of health of older adults [85]. However, the proposed framework was developed to classify mobility indicators that are directly inferable from GPS data. GPS is well suited to investigate an individual’s multi-modal transport patterns—which is inherently referring to different travel speeds of different transport modes [86]. This aspect is represented by the attribute category *transport mode*. Only when zooming further into different transport modes (such as walking), will space–time related categories (such as speed) become interesting to describe a further dimension of individuals’ mobility behaviors. However, such indicators (e.g., walking speed or cadence) typically require higher data quality in order to be assessed reliably, which is possible using camera, pressure sensor or inertial sensor data in controlled, lab-based settings though recently such gait assessments have become possible also in real life using inertial measurement units (IMU) [87].

With respect to transport modes, the most meaningful and most investigated distinction in health and aging research seems to be the distinction between active and passive transport modes as this distinction is required to infer the amount of transport-related physical activity of an individual [46, 47]. Another characteristic of transport mode worth investigating in a healthy aging context would entail the distinction between private and public transport modes. We intentionally limited the framework to aspects derivable from GPS data only and did not include an explicit categorization of further semantic aspects related to an individual’s motives and habits such as trip purpose, experiences along the journey, or company along the travel. However, integrating measurements of social networks and interactions with mobility could be a further way to extend the proposed framework, as social and spatial processes are strongly related [88]. This could be done by combining GPS data with further data sources including self-reports or audio data in order to get insight regarding with and to whom people are traveling [7, 89]. Furthermore, indicators integrating GPS-data with environmental characteristics (e.g., green space, walkability, pollution) to derive exposure measurement are interesting and could be considered as further extensions of the proposed framework. Moreover, future studies might extend the framework by combining the exclusively spatial perspective on mobility of this paper with physical activity indicators, such as active time, sedentary time, and number of steps using IMU sensors to obtain a view of an individual’s daily movement-related patterns beyond the out-of-home spatial activities, including in-home activities [68, 90].

### Underlying dimensions of daily mobility

In order to verify whether indicators reflecting different categories of the classification framework effectively reflect different aspects of an individual’s daily mobility, we applied an exploratory factor analysis on a set of 20 mobility indicators (Table 5) that reflect a comprehensive view of daily mobility according to our framework. The factor analysis revealed the following six factors describing the underlying structure of daily mobility: extent of life space, quantity OH activities, time spent in ATM, stability of life space, elongation of life space, and timing of mobility. A sensitivity analysis showed that the identified dimensions are fairly consistent for different input data requirements (number of included days and minimum hours per day) and across 10 runs to randomize the selection of included study days (Additional file 1: Figures S2–S6).

Some instability in the factor structure was found between the first two factors. For example, location variance appears in factors related to *extent of life space* as well as *quantity of OH activities*. The first factor generally represents more extent-related mobility indicators (which seem to be associated with the amount of traveling using PTM) and is therefore associated with variables reflecting the movement scope *move* or *trajectory*. The second factor depicts the quantity of OH activities, which seems to be associated with *temporal distribution *and overall *duration* of time spent *out of home*. Consequently, this factor draws mainly upon variables related to stops or the entire trajectory. Mobility indicators such as entropy and spatial variance assigned to the category *temporal distribution* were not sufficiently discriminating to form a separate factor, but got intermingled with the first and second factor. However, the variables related to *temporal distribution* in the sense of stability of life space (RevisitedLS, AvgRevisitedLS, SDDirMaxDist) are clearly reflecting a separate dimension of mobility. This is a factor that could have been assigned to the statistical scope *variability*. The factor ‘time spent in active transport modes’ unifies purely indicators reflecting the degree to which individuals use active *transport modes* in their daily mobility. Finally, 'elongation of life space' as well as 'timing of mobility' are two distinct characteristics of an individual’s mobility.

Our findings from the EFA are partially consistent with the few previous studies that have identified the main dimensions of mobility across multiple indicators based on dimension reduction techniques [28, 29, 58]. The dimension *extent of the life space* is consistently found in all the identified approaches (size, action range). Also *elongation of life space* was identified as a characteristic dimension in Sanchez et al. [29] (referred to as circularity) and Perchoux et al. [28]. *Quantity of OH activities* is reflected by what Perchoux et al. [28] labeled volume of activities and Wettstein et al. [58] labeled global out-of-home mobility. The dimension *time spent in ATM* coincides with the dimension coined walking-based mobility by Wettstein and colleagues [58]. Perchoux et al. [28] and Sanchez et al. [29] both identified a further dimension related to time spent in residential neighborhoods and specialization (diversity of activity types), both characteristics that were not represented by our set of mobility indicators as they require additional semantic information on top of GPS data. Truly novel are our two identified factors *timing* and *stability of life space*, which are composed of mobility indicators that, to the best of our knowledge, have not been reported elsewhere so far. In addition, the factor analysis revealed that many of the most frequently used mobility indicators (see Table 2) are reflecting similar properties of the daily mobility. For instance, TOH, maximum distance from home, standard-deviational ellipse, area of convex hull, time in vehicle are all associated with Factor 1.

The indicators we included mostly reflected the average performance (*central tendency*) perspective of mobility. If we had a longer observation period (e.g., a month) and more participants (to have statistically reliable results for a larger number of variables), it would be interesting to include more indicators representing aspects of maximum performance of a participant (e.g., fastest walking speed) or more indicators representing variability (e.g., variability in the number of locations visited). Such indicators could be informed by psychology, in which measures are more established that capture intra-individual variability as an important characteristic that differentiates individuals [60]. Moreover, it would be interesting to test and try to replicate findings for different age populations.

### Implications of this work on health and aging studies

For a holistic view of daily mobility at old age one should ideally assess all identified latent dimensions (results of EFA), as they represent different aspects of mobility. This covers the research gap identified by recent studies (e.g., [91]) which, although recognizing the merits and vast potential of GPS-based mobility assessments, also observe that the disadvantage of such GPS studies is that it is still unclear what meaningful GPS-derived mobility indicators are. In order to represent daily mobility with a minimum set of indicators covering all the identified latent dimensions, we propose a set of representative indicators based on the degree of association with the respective factors (Table 9).

**Table 9:** A minimum set of indicators representing all identified factors

Mobility dimension		Representative indicator
Factor no.	Factor label	
1	Extent of life space	Area of convex hull
2	Quantity OH activities	Number of OH locations
3	Time spent in ATM	Duration in ATM
4	Stability of life space	Percentage revisited area of daily life space
5	Elongation of life space	Gravelius compactness of convex hull
6	Timing of mobility	Time of day at max. distance to home

Representative indicators consist of the indicators that were most associated with the corresponding factors

Future research should aim to identify which dimensions of mobility are important for which outcomes of healthy aging (e.g., active living, independence, social participation). Although the general focus of this paper is on older adults, the proposed framework could be applied to other patient groups that are known to show decreased mobility after a diagnosis or onset of disease (e.g., neurological patients). We expect that depending on the target group and the research questions addressed, not all mobility dimensions will be equally important. *Extent of life space*, for example, could be relevant for the early prediction of cognitive decline, since early-stage dementia patients usually move a lot, however, mostly restricted to their homes [67, 92] due to impaired navigational ability, spatial anxiety etc. *Quantity of OH activities* might be more relevant to assess in people with depressive symptoms since a low number OH activities could be associated with a lower number of social activities [13]. *Time spent in active transport modes* contributes to overall physical activity levels [93, 94] and thus physical health, which should be the focus for sedentary older adults. As mentioned earlier (“Analytical aspects” section), GPS-based variability measures have barely been considered in the health and aging literature. Therefore, it remains to be tested whether stability in life space is positively (in the sense of being constant in behaviors) or negatively (in the sense of less diversity in behaviors) associated with health-beneficial behaviors and health outcomes. *Elongation of life space* could be seen as an indicator reflecting the environment an individual is living in: indicators reflecting higher compactness might be correlated with more urban, dense areas. And finally, *timing of mobility* could again be related to cognitive health. We hypothesize that cognitively healthy individuals would have a more stable circadian rhythm compared to cognitively impaired people. Last but not least, potential future research should examine how different scores along the identified mobility dimensions between individuals are related to aforementioned potential differential health outcomes accounting for other factors shaping individual’s mobility patterns such as the characteristics of the environment that people are exposed to [15, 48].

## Conclusions

GPS tracking is increasingly used in health and aging research to accurately and objectively assess individuals’ mobility in their daily lives. Mobility, however, is a complex concept and it is challenging to characterize it both thoroughly and at the same time also parsimoniously with indicators derived from GPS data.

This paper presents a framework that allows the classification of GPS-based mobility indicators commonly used in literature based on several characteristic and analytical aspects of mobility. Characteristic aspects inform about to the actual semantic properties of a mobility indicator: Is it related to space or time? Which movement scope is concerned and is it enriched with further attributes? Analytical aspects describe how mobility indicators are aggregated and summarized for individuals. The classification scheme aims to demonstrate the breadth of aspects that can be derived from GPS data and to make explicit which aspects are assessed by mobility indicators involved in health and aging studies.

Classifying existing papers that used GPS-derived mobility indicators in health and aging research according to the proposed framework, we found that indicators relating to shape/distribution, timing and temporal distribution of mobility are underrepresented. Consequently we suggest a set of 20 mobility indicators composed of indicators frequently used in the literature, as well as new indicators regarding stability and timing in mobility patterns, with the aim of presenting a comprehensive view of an individual’s daily mobility. Factor analysis based on the 20 suggested mobility indicators confirms that mobility is multi-dimensional and is representable by the six factors: *extent of life space*, *quantity OH activities, time spent in ATM*, *stability of life space*, *elongation of life space*, and *timing of mobility*. Many of the identified factors reflect categories of the suggested classification framework and are, except for the two dimensions *timing of mobility* and *stability of life space*, consistent with the dimensions suggested in previous studies. The framework can be applied for a better understanding of how the different dimensions of mobility relate to healthy aging. This will have implications for clinical practice, informing the development of interventions aiming to enhance daily mobility in old age.

## Additional file

**Additional file 1.** Additional tables and figures presenting (1) detailed classification of exemplary health and aging studies according to the proposed classification framework; (2) summary of identified optimal number of factors for exploratory factor analyses; (3) summary matrices for exploratory factor analyses for the different input data conditions.

## Data Availability

The datasets used and/or analyzed during the current study are available from the corresponding author upon reasonable request.

## Abbreviations

GPS: Global Positioning System
EFA: exploratory factor analysis
Mvt. Sc.: movement scope
Max: maximum
Avg.: average
#: number
%: percent
OH: out of home
ATM: active transport modes
PTM: passive transport modes
MaxDist: maximum distance to home
CHull: area of convex hull
SDE: area of standard deviational ellipse
LocVar: location variance
LengthPerTrip: average trip length
DurPTM: duration in PTM
DurATM: duration in ATM
MaxDurATM: duration of longest ATM trip
NumLoc: number of OH locations
NumUniqLoc: number of unique locations
TOH: time out of home
Entropy: entropy in locations
GravCompact: Gravelius compactness of cHull
Maj2MinAxis: major to minor axis of SDE
RevisitedLS: % revisited area of daily life space
AvgRevisitedLS: avg. % overlapping daily life space
SDDirMaxDist: direction of max. distance fix
TimeMaxDist: time of day at max. distance to home
TimeFirstMove: time of day first move
TimePeriodActive: period of day with most OH activities
M: move
R: random
KMO: Kaiser–Meyer–Okin
BMI: body mass index
