# Supplementary material for: Towards a comprehensive set of GPS-based indicators reflecting the multidimensional nature of daily mobility for applications in health and aging research
Source: Int J Health Geogr. 2019 Jul 24;18:17. doi: 10.1186/s12942-019-0181-0 (PMC6657041; doi:10.1186/s12942-019-0181-0)
Supplement: Supplementary file 1 — Additional file 1. Additional tables and figures presenting (1) detailed classification of exemplary health and aging studies according to the proposed classification framework; (2) summary of identified optimal number of factors for exploratory factor analyses; (3) summary matrices for exploratory factor analyses for the different input data conditions. [file 12942_2019_181_MOESM1_ESM.docx]

Additional File 1

# Detailed classification of relevant health and aging studies

Table S1: Mobility indicators from a selected set of exemplary studies classified according to the characteristic aspects of the mobility indicator classification framework (cf. Figure 2 in the main paper).

| **Study** | **#** | **Mobility indicator** | **Characteristic aspects** | | | | | | | | | | | |
| --- | --- | --- | --- | --- | --- | --- | --- | --- | --- | --- | --- | --- | --- | --- |
|  |  |  | **Space** | | | **Time** | | | **Mvt. sc.** | | | **Attribute** | | |
|  |  |  | **Count** | **Extent** | **Shape/distr.** | **Duration** | **Timing** | **Temp. distr.** | **Stop** | **Move** | **Trajectory** | **Out of home** | **Transport mode** | **Further attribute** |
| Harada et al. (1,2) | 1 | Time out of home (TOH) |  |  |  | ✓ |  |  |  |  | ✓ | ✓ |  |  |
| Kaspar et al. (3) | 2 | TOH |  |  |  | ✓ |  |  |  |  | ✓ | ✓ |  |  |
|  |  | Distance walking |  | ✓ |  |  |  |  |  | ✓ |  |  | ✓ |  |
| Wahl et al. (4) | 2 | TOH |  |  |  | ✓ |  |  |  |  | ✓ | ✓ |  |  |
|  |  | Number of locations | ✓ |  |  |  |  |  | ✓ |  |  |  |  |  |
| Blamoutier et al. (5) | 3 | Time in vehicle |  |  |  | ✓ |  |  |  | ✓ |  |  | ✓ |  |
|  |  | Time on foot |  |  |  | ✓ |  |  |  |  | ✓ |  | ✓ |  |
|  |  | Minimum span ellipse area |  | ✓ |  |  |  |  |  |  | ✓ |  |  |  |
| Cuignet et al. (8) | 3 | Area of standard deviational (SD) ellipse |  | ✓ |  |  |  |  |  |  | ✓ |  |  |  |
|  |  | Time spent in trips |  |  |  | ✓ |  |  |  | ✓ |  |  |  |  |
|  |  | Number of trips | ✓ |  |  |  |  |  |  | ✓ |  |  |  |  |
| Giannouli et al. (6,7) | 4 | Area of convex hull |  | ✓ |  |  |  |  |  |  | ✓ |  |  |  |
|  |  | Mean/max distance from home |  | ✓ |  |  |  |  |  |  | ✓ |  |  |  |
|  |  | Total distance |  | ✓ |  |  |  |  |  |  | ✓ |  |  |  |
| Tung et al. (9) | 4 | Area of convex hull |  | ✓ |  |  |  |  |  |  | ✓ |  |  |  |
|  |  | Perimeter of convex hull |  | ✓ |  |  |  |  |  |  | ✓ |  |  |  |
|  |  | TOH |  |  |  | ✓ |  |  |  |  | ✓ | ✓ |  |  |
|  |  | Mean distance from home |  | ✓ |  |  |  |  |  |  | ✓ | ✓ |  |  |
| Cornwell and Cagney (10) | 5 | Mean distance from home |  | ✓ |  |  |  |  |  |  | ✓ | ✓ |  |  |
|  |  | Area of SD ellipse |  | ✓ |  |  |  |  |  |  | ✓ |  |  |  |
|  |  | Number GPS points outside residential tracts | ✓ |  |  |  |  |  |  |  | ✓ | ✓ |  | ✓ |
|  |  | Number of tracts visited | ✓ |  |  |  |  |  |  |  | ✓ |  |  | ✓ |
|  |  | Number of tracts for at least 10 min | ✓ |  |  | ✓ |  |  |  |  | ✓ |  |  | ✓ |
| Takemoto et al. (11) | 6 | Number of trips on foot / in vehicle | ✓ |  |  |  |  |  |  | ✓ |  |  | ✓ |  |
|  |  | Distance on foot / in vehicle |  | ✓ |  |  |  |  |  | ✓ |  |  | ✓ |  |
|  |  | Time on foot / in vehicle |  |  |  | ✓ |  |  |  | ✓ |  |  | ✓ |  |
| Wettstein et al. (12, 13, 14*, 15**) | 5, 7*, 8** | TOH |  |  |  | ✓ |  |  |  |  | ✓ | ✓ |  |  |
|  |  | Number of locations | ✓ |  |  |  |  |  | ✓ |  |  |  |  |  |
|  |  | Distance on foot |  | ✓ |  |  |  |  |  | ✓ |  |  | ✓ |  |
|  |  | Time on foot |  |  |  | ✓ |  |  |  | ✓ |  |  | ✓ |  |
|  |  | Walking speed |  |  |  |  |  |  |  | ✓ |  |  | ✓ | ✓ |
|  |  | Walking duration per track* |  |  |  | ✓ |  |  |  | ✓ |  |  | ✓ |  |
|  |  | Number of walking tracks*^/^** | ✓ |  |  |  |  |  |  | ✓ |  |  | ✓ |  |
|  |  | Mean/max distance from home** |  |  |  |  |  |  |  |  | ✓ |  |  |  |
| Boissy et al. (16) | 10 | Time at home |  |  |  | ✓ |  |  |  |  | ✓ | ✓ |  |  |
|  |  | Number of trips | ✓ |  |  |  |  |  |  | ✓ |  |  |  |  |
|  |  | Number of locations | ✓ |  |  |  |  |  | ✓ |  |  | ✓ |  |  |
|  |  | Maximum distance from home |  | ✓ |  |  |  |  |  |  | ✓ | ✓ |  |  |
|  |  | Distance in vehicle |  | ✓ |  |  |  |  |  | ✓ |  |  | ✓ |  |
|  |  | Time in vehicle |  |  |  | ✓ |  |  |  | ✓ |  |  | ✓ |  |
|  |  | Time on foot |  |  |  | ✓ |  |  |  | ✓ |  |  | ✓ |  |
|  |  | Distance on foot |  | ✓ |  |  |  |  |  | ✓ |  |  | ✓ |  |
|  |  | Minimum span ellipse major axis length |  | ✓ |  |  |  |  |  |  | ✓ |  |  |  |
|  |  | Minimum span ellipse area |  | ✓ |  |  |  |  |  |  | ✓ |  |  |  |
| Saeb et al. (17,18) | 11 | Location variance |  |  | ✓ |  |  |  |  |  | ✓ |  |  |  |
|  |  | Circadian movement |  |  |  |  | ✓ |  |  |  | ✓ |  |  |  |
|  |  | Speed mean |  |  |  |  |  |  |  |  | ✓ |  |  | ✓ |
|  |  | Speed variance |  |  |  |  |  |  |  |  | ✓ |  |  | ✓ |
|  |  | Distance |  | ✓ |  |  |  |  |  |  | ✓ |  |  |  |
|  |  | Number of locations | ✓ |  |  |  |  |  | ✓ |  |  |  |  |  |
|  |  | Entropy |  |  |  |  |  | ✓ | ✓ |  |  |  |  |  |
|  |  | Normalized entropy |  |  |  |  |  | ✓ | ✓ |  |  |  |  |  |
|  |  | Raw entropy |  |  |  |  |  | ✓ | ✓ |  |  |  |  |  |
|  |  | Time at home |  |  |  | ✓ |  |  |  |  | ✓ | ✓ |  |  |
|  |  | Percentage time traveling |  |  |  | ✓ |  |  |  | ✓ |  | ✓ |  |  |
| Isaacson et al. (19) | 11 | TOH |  |  |  | ✓ |  |  |  |  | ✓ | ✓ |  |  |
|  |  | Number of locations | ✓ |  |  |  |  |  | ✓ |  |  |  |  |  |
|  |  | Duration on foot |  |  |  | ✓ |  |  |  | ✓ |  |  | ✓ |  |
|  |  | Number of trips on foot | ✓ |  |  |  |  |  |  | ✓ |  |  | ✓ |  |
|  |  | Time in trips |  |  |  | ✓ |  |  |  | ✓ |  |  | ✓ |  |
|  |  | Number of trips in vehicle | ✓ |  |  |  |  |  |  | ✓ |  |  | ✓ |  |
|  |  | Average time per trip in vehicle |  |  |  | ✓ |  |  |  | ✓ |  |  | ✓ |  |
|  |  | Average time per trip on foot |  |  |  | ✓ |  |  |  | ✓ |  |  | ✓ |  |
|  |  | Average distance per trip on foot |  | ✓ |  |  |  |  |  | ✓ |  |  | ✓ |  |
|  |  | Driving speed |  |  |  |  |  |  |  | ✓ |  |  | ✓ | ✓ |
|  |  | Walking speed |  |  |  |  |  |  |  | ✓ |  |  | ✓ | ✓ |
| Sanchez et al. (20) | 25 | Percentage time spent in 50/100/400/800/1600 m residential buffers |  |  |  | ✓ |  |  |  |  | ✓ | ✓ |  | ✓ |
|  |  | Percentage time spent in village boundaries |  |  |  | ✓ |  |  |  |  | ✓ | ✓ |  | ✓ |
|  |  | Percentage time spent at home |  |  |  | ✓ |  |  |  |  | ✓ | ✓ |  |  |
|  |  | Percentage time spent at OH locations |  |  |  | ✓ |  |  | ✓ |  |  | ✓ |  |  |
|  |  | Percentage time spent in trips |  |  |  | ✓ |  |  |  | ✓ |  |  |  |  |
|  |  | Number OH locations | ✓ |  |  |  |  |  | ✓ |  |  | ✓ |  |  |
|  |  | Percentage of locations inside village boundaries | ✓ |  |  |  |  |  | ✓ |  |  | ✓ |  | ✓ |
|  |  | Percentage of locations inside SD ellipse |  | ✓ | ✓ |  |  |  | ✓ |  | ✓ | ✓ |  |  |
|  |  | Average distance from home to locations |  | ✓ |  |  |  |  | ✓ |  |  | ✓ |  |  |
|  |  | Perimeter/surface of convex hull / SD ellipse |  | ✓ |  |  |  |  |  |  | ✓ |  |  |  |
|  |  | Compactness of convex hull / SD ellipse |  |  | ✓ |  |  |  |  |  | ✓ |  |  |  |
|  |  | Distance from home to centroid of convex hull / SD ellipse |  | ✓ | ✓ |  |  |  |  |  | ✓ | ✓ |  |  |
|  |  | Mean/median distance from home |  | ✓ |  |  |  |  |  |  | ✓ | ✓ |  |  |

Note. ‘Mvt. sc.’ is abbreviated for ‘Movement scope’. ‘#’ refers to the number of GPS-based mobility indicators used in the study. Multiple studies published by the same first author involving a similar set of mobility indicators are counted as one study. The underlined reference is the one we used for the other tables building on this one (i.e., Table 1 and Table 2 in the main paper). The studies are ordered by increasing number of included GPS-based mobility indicators.

# Optimal number of factors for exploratory factor analyses across all runs

Figure S1: Optimal number of factors according to the 4 statistical tests (Kaiser Rule, Parallel Analysis, Optimal Coordinates, Acceleration Factor) across the 10 runs of all the 5 input data conditions, adding up to 2000 suggested factor number solutions.

# Summary matrices for exploratory factor analyses for different inclusion criteria


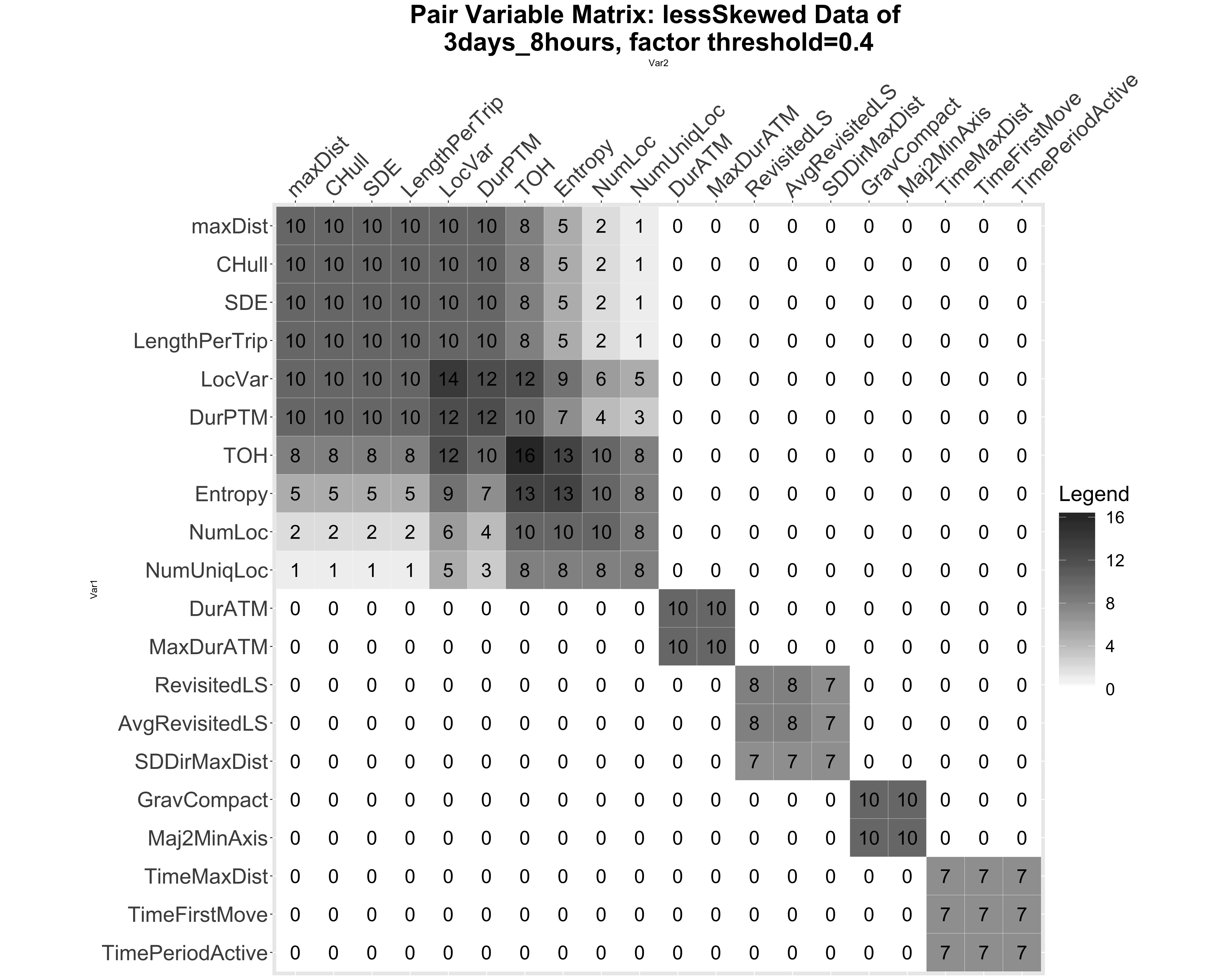


Figure S2: Exploratory factor analysis (EFA) summary matrix for the 10 runs of random day selection, with the inclusion criteria 3 days of at least 8 hours per participant.


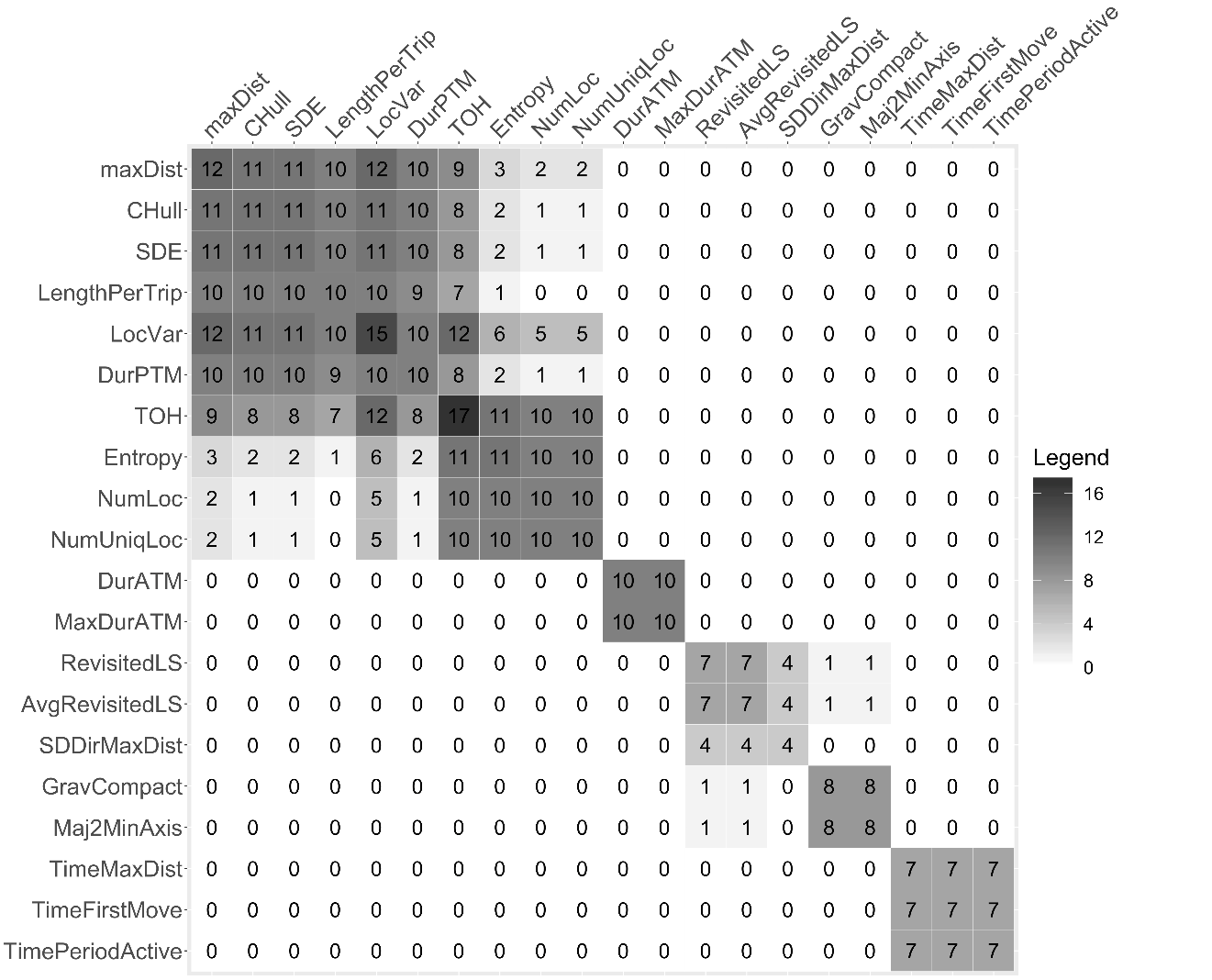


Figure S3: EFA summary matrix for the 10 runs of random day selection, with the inclusion criteria 4 days of at least 8 hours per participant.


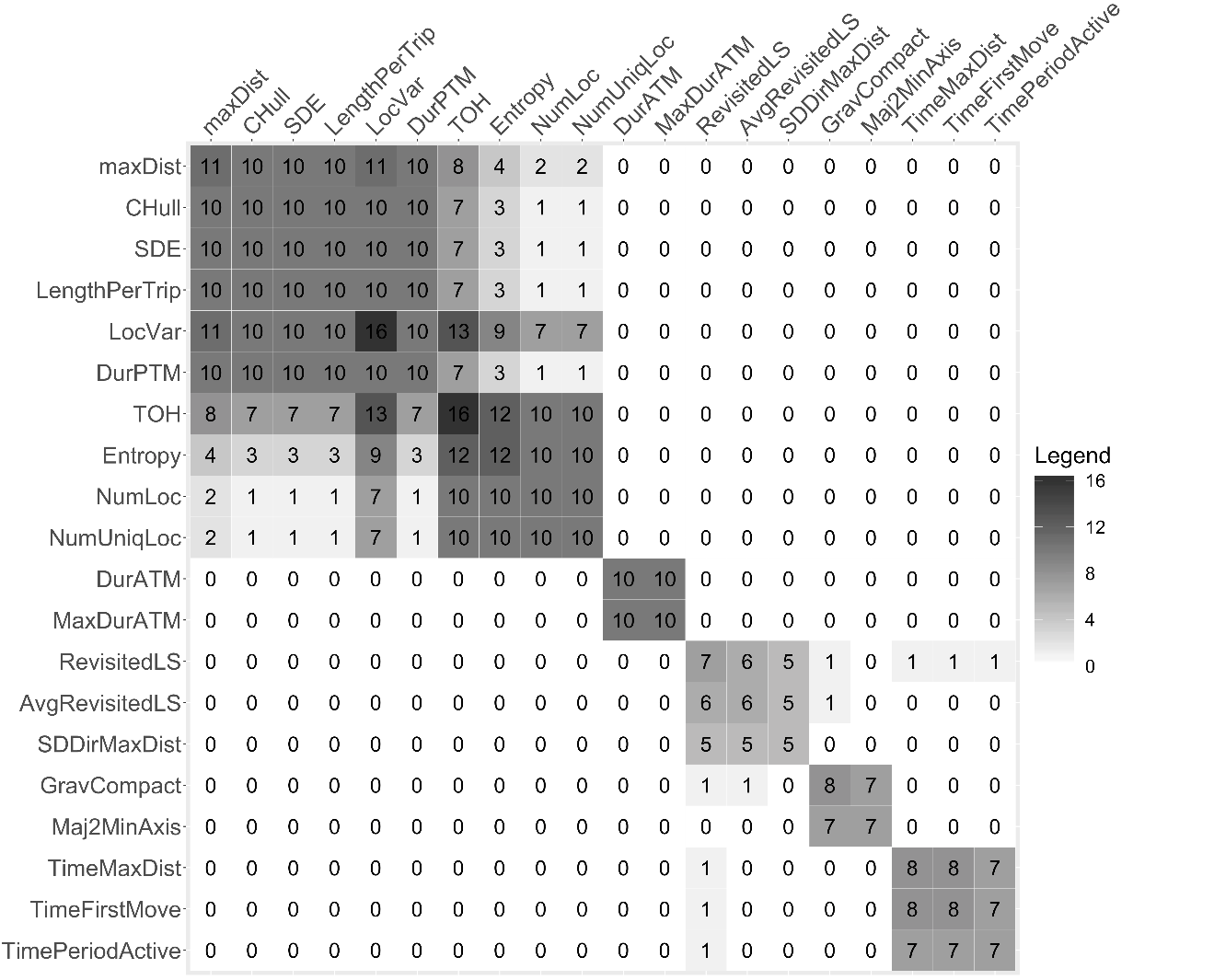


Figure S4: EFA summary matrix for the 10 runs of random day selection, with the inclusion criteria 4 days of at least 9 hours per participant.


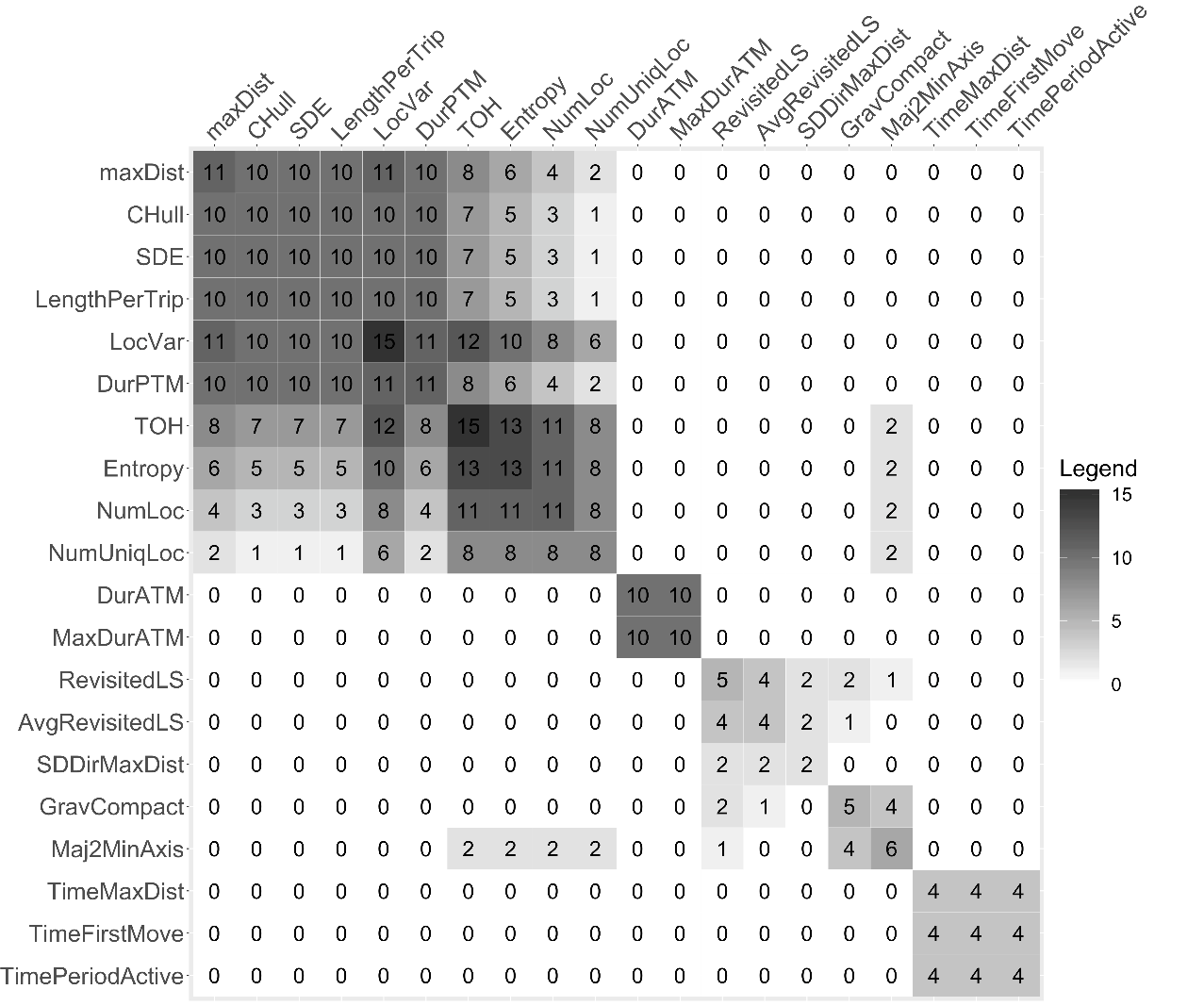


Figure S5: EFA summary matrix for the 10 runs of random day selection, with the inclusion criteria of 4 days with at least 10 hours per participant.


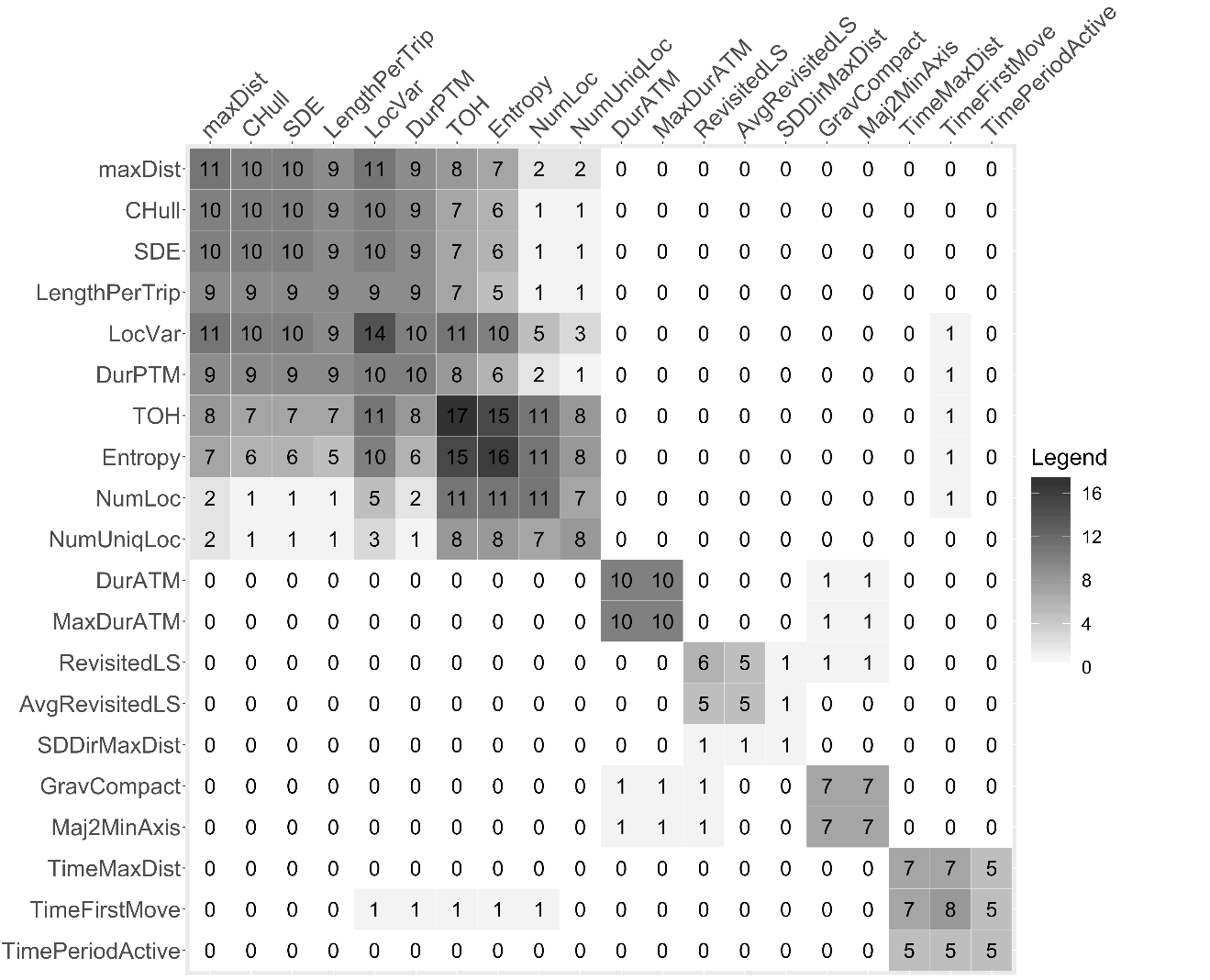


Figure S6: EFA summary matrix for the 10 runs of random day selection, with the inclusion criteria 5 days with at least 10 hours per participant.

References for Additional File 1

1. Harada K, Lee S, Lee S, Bae S, Harada K, Suzuki T, et al. Objectively-measured outdoor time and physical and psychological function among older adults. Geriatrics and Gerontology International. 2017;17(10):1455–62.

2. Harada K, Lee S, Lee S, Bae S, Harada K, Shimada H. Changes in objectively measured outdoor time and physical, psychological, and cognitive function among older adults with cognitive impairments. Archives of Gerontology and Geriatrics [Internet]. 2018 Sep 1 [cited 2019 Jan 24];78:190–5. Available from: https://www.sciencedirect.com/science/article/pii/S0167494318301171

3. Kaspar R, Oswald F, Wahl H-W, Voss E, Wettstein M. Daily mood and out-of-home mobility in older adults: does cognitive impairment matter? Journal of Applied Gerontology [Internet]. 2015 Feb;34(1):26–47. Available from: http://jag.sagepub.com/cgi/doi/10.1177/0733464812466290

4. Wahl H-W, Wettstein M, Shoval N, Oswald F, Kaspar R, Issacson M, et al. Interplay of cognitive and motivational resources for out-of-home behavior in a sample of cognitively heterogeneous older adults: findings of the SenTra project. Journals of Gerontology Series B: Psychological Sciences and Social Sciences. 2013;68(5):691–702.

5. Blamoutier M, Boissy P, Brière S, Faucher G, Lauzé M, Duval C. Is a decrease of grip strength associated with community mobility restriction in dynapenic older women? JCSM Clinical Reports [Internet]. 2018;3(1):1–9. Available from: https://jcsm-clinical-reports.info/index.php/jcsm-cr/article/view/42

6. Giannouli E, Bock O, Mellone S, Zijlstra W. Mobility in old age: capacity is not performance. BioMed Research International [Internet]. 2016;(February):1–8. Available from: http://www.hindawi.com/journals/bmri/2016/3261567/

7. Giannouli E, Bock O, Zijlstra W. Cognitive functioning is more closely related to real-life mobility than to laboratory-based mobility parameters. European Journal of Ageing. 2018;1–9.

8. Cuignet T, Perchoux C, Caruso G, Klein O, Klein S, Chaix B, et al. Mobility among older adults: deconstructing the effects of motility and movement on wellbeing. Urban Studies [Internet]. 2019; Available from: https://journals.sagepub.com/doi/pdf/10.1177/0042098019852033

9. Tung JY, Rose RV, Gammada E, Lam I, Roy EA, Black SE, et al. Measuring life space in older adults with mild-to-moderate Alzheimer’s disease using mobile phone GPS. Gerontology. 2014;60:154–62.

10. Cornwell EY, Cagney KA. Aging in activity space: results from smartphone-based GPS-tracking of urban seniors. Journals of Gerontology: Social Sciences. 2017;72(5):864–75.

11. Takemoto M, Carlson JA, Moran K, Godbole S, Crist K, Kerr J. Relationship between objectively measured transportation behaviors and health characteristics in older adults. International Journal of Environmental Research and Public Health. 2015;12(11):13923–37.

12. Wettstein M, Wahl HW, Diehl MK. A multidimensional view of out-of-home behaviors in cognitively unimpaired older adults: examining differential effects of socio-demographic, cognitive, and health-related predictors. European Journal of Ageing. 2014;11(2):141–53.

13. Wettstein M, Wahl H-W, Shoval N, Oswald F, Voss E, Seidl U, et al. Out-of-home behavior and cognitive impairment in older adults: findings of the SenTra project. Journal of Applied Gerontology [Internet]. 2015;34(1):3–25. Available from: http://jag.sagepub.com/cgi/doi/10.1177/0733464812459373

14. Wettstein M, Wahl HW, Shoval N, Auslander G, Oswald F, Heinik J. Identifying mobility types in cognitively heterogeneous older adults based on GPS-tracking: what discriminates best? Journal of Applied Gerontology. 2015;34(8):1001–27.

15. Wettstein M, Wahl HW, Shoval N, Auslander G, Oswald F, Heinik J. Cognitive status moderates the relationship between out-of-home behavior (OOHB), environmental mastery and affect. Archives of Gerontology and Geriatrics [Internet]. 2014;59(1):113–21. Available from: http://dx.doi.org/10.1016/j.archger.2014.03.015

16. Boissy P, Blamoutier M, Brière S, Duval C. Quantification of free-living community mobility in healthy older adults using wearable sensors. Frontiers in Public Health [Internet]. 2018;6:1–13. Available from: https://www.frontiersin.org/article/10.3389/fpubh.2018.00216/full

17. Saeb S, Lattie EG, Schueller SM, Kording KP, Mohr DC. The relationship between mobile phone location sensor data and depressive symptom severity. PeerJ. 2016;4:e2537.

18. Saeb S, Zhang M, Karr CJ, Schueller SM, Corden ME, Kording KP, et al. Mobile phone sensor correlates of depressive symptom severity in daily-life behavior: an exploratory study. Journal of Medical Internet Research [Internet]. 2015 Jul 15;17(7):e175. Available from: http://www.jmir.org/2015/7/e175/

19. Isaacson M, Wahl H, Shoval N, Oswald F, Auslander G. The relationship between spatial activity and wellbeing-related data among healthy older adults: an exploratory geographic and psychological analysis. In: Samanta T, editor. Cross-Cultural and Cross-Disciplinary Perspectives in Social Gerontology [Internet]. Singapore: Springer Singapore; 2017. p. 203–19. Available from: http://link.springer.com/10.1007/978-981-10-1654-7

20. Sanchez M, Ambros A, Salmon M, Bhogadi S, Wilson RT, Kinra S, et al. Predictors of daily mobility of adults in peri-urban south India. International Journal of Environmental Research and Public Health. 2017;14(7):1–16.
